# Sexual Neurasthenia

**Published:** 1884-10

**Authors:** 


**Sexual Neurasthenia:** Its Hygienic Causes, Symptoms and Treatment, with a Chapter on Diet for the Nervous. By GEORGE M. BEARD, M. D., formerly Lecturer on Nervous Diseases, University City of New York, etc. Edited by A. D. ROCKWELL, A. M., M. D. New York: E. B. Treat, 757 Broadway. 1884. Price, $2.00.

This work was in manuscript at the untimely taking off of its brilliant author. His confrere has ably edited the work, and it is one which may be read with advantage by all practitioners. The honesty and fearlessness of its author, as well as the purity of his character, fitted him for the consideration of the delicate but important topics herein discussed, and the subject is presented in such a clear and lively way as to compel attention. While we cannot always agree with his conclusions, still the work is eminently suggestive, sparkling with originality, and its perusal will repay the physician.

